# Prognostic Value of Affective Symptoms in First-Admission Psychotic Patients

**DOI:** 10.3390/ijms17071039

**Published:** 2016-06-30

**Authors:** Marta Arrasate, Itxaso González-Ortega, Adriana García-Alocén, Susana Alberich, Iñaki Zorrilla, Ana González-Pinto

**Affiliations:** 1 RSMB-Algorta-Vizcaya, Osakidetza, University of the Basque Country (EHU/UPV), Leioa 48940, Vizcaya, Spain; 2 CIBERSAM, BIOARABA Health Research Institute, OSI Araba, Department of Psychiatry, Araba University Hospital-Santiago, University of the Basque Country (EHU/UPV), Vitoria 01004, Alava, Spain; itxaso.gonzalezortega@osakidetza.eus (I.G.-O.); adriana.garciaalocen@osakidetza.eus (A.G.-A.); susana.alberichmesa@osakidetza.eus (S.A.); inaki.zorrillamartinez@osakidetza.eus (I.Z.); anamaria.gonzalez-pintoarrillaga@osakidetza.eus (A.G.-P.)

**Keywords:** prognostic, affective, dimension, first-episode psychosis

## Abstract

Background: Very little research has been conducted in patients with first-episode psychosis using a dimensional approach. Affective dimensional representations might be useful to predict the clinical course and treatment needs in such patients. Methods: Weincluded 112 patients with first-episode psychosis in a longitudinal-prospective study with a five-year follow-up (*N* = 82). Logistic analyses were performed to determine the predictive factors associated with depressive, manic, activation, and dysphoric dimensions. Results: High scores on the depressive dimension were associated with the best prognosis. On the other hand, high scores on the activation dimension and the manic dimension were associated with a poorer prognosis in terms of relapses. Only the dysphoric dimension was not associated with syndromic or functional prognosis. Conclusion: Ourresults suggest that the pattern of baseline affective symptoms helps to predict the course of psychotic illness. Therefore, the systematic assessment of affective symptoms would enable us to draw important conclusions regarding patients’ prognosis. Interventions for patients with high scores on manic or activation dimensions could be beneficial in decreasing relapses in first-episode psychosis.

## 1. Introduction

The term first-episode psychosis is applied to a heterogeneous population with a wide range of diagnoses. The clinical definition of psychosis may encompass only one part of the broad phenotypic spectrum [1]. Today’s classification systems are increasingly more focused on dimensions besides categories to describe diagnoses in psychiatry.

Additionally, the influence of affective symptoms on functional psychosis has been studied little, and results are frequently controversial. Moreover, there has been minimal research in this field focusing on first psychotic episodes, and only a few studies have used a dimensional approach. Nevertheless, it is plausible that dimensional representations would be useful to predict the clinical course and treatment needs of patients in first-episode psychosis.

Crow [2] and van Os [3] suggested the continuum hypothesis in which different diagnostic groups share dimensional factors, which could refer to similar neurobiological mechanisms, regardless of the type of psychosis. These dimensions in psychosis are not diagnosis-specific and seem to be reasonably replicable and stable in a variety of settings, diagnostic groups, and patient samples [4]. Early work was done on schizophrenia, finding a three-factor solution, with positive, negative, and disorganized dimensions [5]. Some later studies, those of Cassidy [6], Serretti [7], and Disalver [8], examined the factor structure of bipolar disorder, finding four factors (including depression, mania, insomnia, and dysphoria), while, González-Pinto et al. [9] obtained a five-factor solution in individuals with this diagnosis. More recently, samples studied have included the full spectrum of psychosis, and five-factor solutions have again been found, including manic and depressive dimensions [10,11,12,13,14,15]. In parallel, factor structure analyses have also focused on first psychotic episode samples [4,16,17,18,19,20,21] finding a five-factor solution, with positive, negative, manic, depressive, and disorganization dimensions.

Regarding the influence of affective symptoms in psychosis, some authors found they are associated with a good prognosis [17,22,23,24,25], some relating the better prognosis specifically to higher depressive dimension scores [19,26]. Further, both van Os et al. [17] and Allardyce et al. [27] associated higher scores in the manic dimension with a good outcome; the former study identifying that patients had fewer and less severe symptoms and the latter that patients were more likely to be married and working.

In contrast, others researchers have found a negative association between depression and outcome: Geddes et al. [28] identified early relapse and more time in hospital, Birchwood [29] also observed early relapse, and Meng et al. [30] cited a poor prognosis. Further, Thara et al. [31] associated a longer history of symptoms with manic decompensation. Affective symptoms were found to be associated with more hospitalizations by Power et al. [32] and with a poor outcome by Sipos et al. [33].

In this context, our objective was to study the predictive value of affective symptoms in patients followed up at three and five years after their first episode of psychosis using a dimensional approach. We studied outcome in terms of hospitalization, relapses, and suicidal behaviour.

## 2. Results

### 2.1. Patient Sociodemographic and Clinical Characteristics

A total of 112 patients with a first psychotic episode were included in the study at baseline. Of these patients, 91 (81.25%) and 82 (73.2%) patients were available for analysis at three and five years of follow-up. Sociodemographic and clinical baseline characteristics have been described in a previous publication [34].

There were no differences between patients who were and were not followed up at Year 3 or Year 5 (the results at Year 5 with respect to baseline were as follows: *U* = 1023, *p* = 0.62 for age; χ^2^ = 0.30, *p* = 0.58 for sex; Fisher’s exact *p* values of 0.69 for marital status, 0.27 for socioeconomic level and 0.53 for smoking).

### 2.2. Affective Dimensions

As previously described, factor structure analysis [9] identified a five-factor solution, and this explained 60.8% of the total variance in a sample of 103 patients with bipolar disorder. In the present study, we analysed four of these affective dimensions. The depressive dimension included symptoms of depressed mood, suicidal thoughts, feelings of guilt, obsessive and compulsive symptoms, and anxiety, and patients obtained a mean score of 3.92 (SD = 3.65) at baseline. The *dysphoric dimension* included disruptive-aggressive behaviour, irritability, and lack of insight, and patients obtained a mean score of 8.55 (SD = 4.89) at baseline. The *manic dimension* included appearance, sexual interest, elevated mood, and reduced sleeping, and patients obtained a mean score of 5.27 (SD = 3.44) at baseline. Finally, the *activation dimension* included speech being difficult to understand, increased motor activity-energy and language-thought disorder, and patients obtained a mean score of 5.37 (SD = 4.78) at baseline.

### 2.3. Clinical Characteristics at Follow-up

Of the 91 patients assessed at Year 3, 80.2% had relapses, 61.5% hospitalizations, and 19.8% suicide attempts during the follow-up. Of the 82 assessed at Year 5, 91.46% had relapses, 73.17% hospitalizations, and 21% suicide attempts over the course of the entire follow-up period.

### 2.4. Diagnostic Predictive Value of Affective Dimensions

The predictive value of affective symptoms was determined by analysing the influence of dimensions on hospitalizations, relapses, and suicidal behaviour using regression models.

With respect to the depressive dimension, we observed that higher scores were significantly associated with fewer relapses at Year 5 and fewer hospitalizations at Year 3 (β = −0.03: 95% CI: 0.94 to 0.99, *p* = 0.045 and β = −0.08, 95% CI: 0.87 to 0.98, *p* = 0.012), while higher scores in the manic dimension were significantly associated with more relapses (β = 0.04, 95% CI: 1.01 to 1.08, *p* = 0.023) at Year 5. Further, higher scores in the activation dimension were significantly associated with the presence of relapses at Year 3 (OR: 1.13; 95% CI: 1 to 1.27, *p* = 0.050) and a higher number of relapses (β = 0.03, 95% CI: 1.01 to 1.06, *p* = 0.016) at Year 5. In contrast, the dysphoric dimension was not significantly associated with any of the variables tested (Table 1).

## 3. Discussion

This prospective, longitudinal study of the diagnostic predictive value of affective symptoms in a sample of patients hospitalized for first-episode psychosis and followed-up over five years shows that different affective dimensions (manic, activation, dysphoric, and depressive) have a different influence on prognosis in psychosis.

Regarding relapses, we found high rates of 80.2% to 91.46%, similar to that of Robinson et al. [35] (86.2% at Year 5), but somewhat higher than the range reported by other authors (58%–78%) [29,36,37,38]. The difference may be attributable to the diverse definitions used for “relapse”; in addition, our patients were hospitalized and had more severe psychosis. In our study, higher manic and activation dimension scores were associated with more relapses, while high scores in the depressive dimension were protective against relapse.

With respect to hospitalizations, 61.5% of the sample was hospitalized at some point in the first three years, and 73.17% had been hospitalized by the end of the five-year follow-up period—consistent with the findings of Power et al. [32]. Means are similar for the two periods and very similar to those reported by Sipos et al. [33]. In contrast, Swaran et al. [25] found higher rates. Admission rates depend on a variety of factors, in particular, organization of both in- and out-patient mental health services and access. In our study, higher scores in the depressive dimension protected against hospitalization.

With regard to suicide, 19.8% of patients had attempted suicide by Year 3, rising to 21% by Year 5, percentages that are in agreement with data from Birchwood et al. [29], van Os et al. [17], Verdoux et al. [39], and Robinson et al. [35], and the mean was similar in the two periods. Two patients committed suicide in the last two years (2.4%); unfortunately, as Birchwood noted, suicide risk does not necessarily fall after the first years of the illness [29,40,41].

Concerning the prognostic value of affective dimensions, higher scores in the depressive dimension were significantly associated with fewer hospitalizations and relapses at Years 3 and 5, respectively, and hence with a good prognosis. Many authors have reported a better outcome [14,19,25,26,42] in the presence of depressive symptoms. On the other hand, we should note that some authors have not found an association between the depressive dimension and outcome [17,30,37,43,44], and others have described a poorer course [10,28,31,45], while Lindenmayer and Kay [46] question the role of negative symptoms in unfavourable outcomes. We took into account the possible effect of negative symptoms, controlling for baseline symptoms in our statistical analyses. Further, Peralta et al. [5] found that the depressive dimension was associated with negative factors. Given this, we used assessment tools, which are specifically designed for rating affective rather than negative symptoms.

Higher manic dimension scores were significantly associated with more relapses by the end of the follow-up period. Similarly, higher activation dimension scores were associated with relapse by Year 3 and more relapses by Year 5. That is, the higher the activation dimension score, the higher the risk of relapse. In turn, high scores in manic and activation dimensions are related to a poorer symptomatic outcome. Consistent with our findings, Sipos et al. [33] and Gift et al. [44] indicate that high manic dimension scores carry a high risk of hospitalization. In contrast, Murray et al. [14], McIntosh et al. [19], and van Os et al. [17] described a better symptomatic outcome.

Finally, the dysphoric dimension was not associated with any of the variables described above.

In summary, only one of the dimensions considered does not seem to be associated with syndromic prognosis, namely, the dysphoric dimension. High scores in the depressive dimension are linked to the best prognosis. In contrast, high scores in the activation dimension, in general, give an unfavourable prognosis in terms of relapses, as do high scores in the manic dimension.

Our results suggest that the pattern of affective symptoms present does help to predict the course of psychotic illness. Therefore, the systematic evaluation of affective symptoms may enable us to draw important conclusions regarding patients’ prognosis. Moreover, adding mood stabilizers to the treatment of patients with manic or activation syndromes could be beneficial in reducing relapses in first-episode psychosis.

We would like to underline that the main contribution of this research is the application of affective dimensions established in a bipolar disorder sample by a factor structure analysis to a sample of patients with functional psychosis to explore the prognostic value of the dimensions identified. We also would like to emphasize the representativeness of the sample, as our unit is the only one for acute inpatients in our region. In addition, our study is longitudinal with a long follow-up of a heterogeneous sample.

Nevertheless, some limitations must be considered. First, a number of patients were taking medication; we attempted to minimise the impact of this by assessing them within 24 h of hospitalization. Secondly, patients with more severe conditions are probably overrepresented; thus, the generalization of the results is limited to patients who are hospitalized. Nevertheless, more than 80% of patients with first psychotic episodes are hospitalized. In addition, a few of the assessments were made by telephone, when patients were not able to attend appointments. Finally, the main limitation is that we have not adjusted results for drug use; cannabis use is frequent in this kind of patient and is known to have an influence on psychotic episodes. Therefore, we will explore this issue in future studies. On the other hand, we did adjust for age, sex, negative symptoms, and premorbid adjustment following widely used methods.

Despite these limitations, our results strongly suggest that affective symptoms influence the prognosis of psychosis and hence that systematic evaluation of affective symptoms would provide us with some useful information about prognosis. Further, intervention in patients with manic or activation syndrome could be beneficial in minimizing relapses in patients with first-episode psychosis. We suggest making a more specific assessment, attempting to confirm the presence, or absence, of manic and activation dimensions, to guide the provision of more comprehensive treatment. The fact that these results were obtained after controlling the analyses for the presence of negative symptoms and premorbid adjustment and, therefore, baseline functionality, enhances the reliability of the findings.

## 4. Materials and Method

### 4.1. Study Design and Participants

One hundred and twelve patients presenting with a first episode of psychosis between January 1996 and December 1997, and who were admitted to the only psychiatric inpatient unit in the Vitoria-Gasteiz region of Spain were included in a prospective, longitudinal study. First episode psychosis was defined as the first time a patient presented with psychotic symptomatology: delusions, hallucinations, grossly disorganized behaviour, marked thought disorder, or any combination of these symptoms.

Patients were included in the study if they met the following inclusion criteria: aged between 16 and 65 years and meeting diagnostic criteria of the Fourth Edition of the Diagnostic and Statistical Manual of Mental Disorders (DSM-IV) [47] for schizophreniform disorder, schizoaffective disorder, schizophrenia, delusional disorder, brief psychotic disorder, atypical psychosis, or psychotic disorder not otherwise specified, bipolar I or II disorder, or major depressive disorder with psychotic symptoms. The DSM-IV axis I diagnosis was made using the Structured Clinical Interview for DSM-IV (SCID-I) [48]; the same interviewers conducted baseline and follow-up assessments. Individuals with mental retardation, organic brain disorders, or substance-induced psychotic disorders as their main diagnosis were excluded from the study.

The study was approved by the ethics committee of the hospital, and all participants gave written informed consent.

### 4.2. Assessments

Patients were assessed at baseline and at 3 and 5 years of follow-up within 24 h of hospitalization for the first psychotic episode and reflected the patient’s clinical status during the previous week.All patients assessed during the following-period were in treatment.

Data collected included patient sociodemographic and clinical characteristics. Patients were assessed by different raters from those who made the diagnosis using the following scales: Young Mania Rating Scale (YMRS) [49], Hamilton Depression Rating Scale (HDRS-21) [50,51], Phillips Rating Scale of Premorbid Adjustment in Schizophrenia (Phillips) [52], and the Positive and Negative Syndrome Scale (PANSS) [53]. All interviews were carried out independently by one psychiatrist and one psychologist who demonstrated good inter-rater reliability for SCID diagnoses (κ = 0.88), and YMRS (κ = 0.90), HDRS-21 (κ = 0.93), Phillips (κ = 0.80), and PANSS (κ = 0.82) scores.

The choice of affective dimensions was based on a previous factor structure analysis using the YMRS and HDRS-21 in 103 patients with bipolar disorder. This gave a five-factor solution and the component symptom loadings obtained for each of the affective dimensions (depressive, dysphoric, manic, psychosis, and activation) are summarised in a previous publication [9]. Factor structure analysis has been widely used for research purposes and in clinical trials for studying the symptom dimensions of psychosis [4,11,12,14,16,17,18,19,33]. In the present study, we analysed baseline scores for four affective dimensions (depressive, dysphoric, manic, and activation); the psychosis factor was not used because all patients presented with psychotic symptoms.

### 4.3. Statistical Analysis

Statistical packages used for the analyses were SPSS v23 and R 2.5.1 (R Core Team, Viena, Austria) [54].

Baseline characteristics of the sample were described using means and standard deviations (SD) or median and range, for continuous variables, depending on their normality and frequencies for categorical variables.

The prognostic value of affective dimensions was examined using regression models, with number of hospitalizations, relapses and suicidal behaviour as the dependent variables. Relapse was defined as the presence of another clinical episode indicated by SCID score (≥3 in a variety of items) and meeting the DSM-IV clinical criteria for active illness according to data in the patient’s medical record. A logistic regression model including all four affective dimensions as independent variables was used to identify which dimensions were predictive of the evolution of first-admission psychotic patients. Logistic regressions were adjusted for age and sex, negative symptoms (PANSS-N), and premorbid state (Phillips Rating Scale of Premorbid Adjustment) according to the method used by other researchers [46], since it is known these variables influence the outcome. Effect sizes are expressed as odds ratios (ORs) and 95% confidence intervals (CIs) with *p* values. Effect sizes estimated from Poisson regressions are expressed as β coefficients, and 95% CIs with *p* values. Associations were considered significant when *p* ≤ 0.05.

## 5. Conclusions

Clearly, there is no reason why changes in the variables studied should reflect the general course of the illness as was clarified through a study of illness course by the World Health Organization [55]; the variables that determine the global course are different and varied. This affirmation is in harmony with the idea that it is necessary to differentiate between syndromic and functional recovery. One must bear in mind that this differentiation is valuable when proposing strategies for the prevention of relapses and improving the prognosis of patients with real possibilities of recovery.

## Figures and Tables

**Table 1:** Functional outcomes at 3 and 5 years of follow-up.

Dimension	Year 3	Year 5
Depressive	Higher depressive dimension scores, fewer hospitalizations (β = −0.08, 95% CI: 0.87 to 0.98, *p* = 0.012)	Higher depressive dimension scores, fewer relapses (β = −0.03: 95% CI: 0.94 to 0.99, *p* = 0.045)
Manic	-	Higher manic dimension scores, more relapses (β = 0.04, 95% CI: 1.01 to 1.08, *p* = 0.023)
Activation	Presence of relapses (OR: 1.13; 95% CI: 1 to 1.27, *p* = 0.050)	Higher activation dimension scores, more relapses (β = 0.03, 95% CI: 1.01 to 1.06, *p* = 0.016)
Dysphoric	-	-
